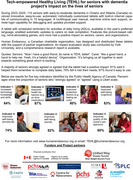# Environmental design and technology for people living with dementia

**DOI:** 10.1002/alz70861_108713

**Published:** 2025-12-23

**Authors:** Noor Din, Lois Kamenitz

**Affiliations:** ^1^ Human Endeavour Incorporation, Woodbridge, ON Canada; ^2^ York University, Toronto, ON Canada

## Abstract

**Background:**

Innovative assistive technology is redefining care for seniors with dementia and mild cognitive impairment (MCI), offering a paradigm shift that enhances the well‐being of both seniors and carers. It also enables service enhancements beyond what traditional in‐person care can provide. Yet, current off‐the‐shelf solutions remain inadequate for meeting the unique and complex needs of this population.

**Method:**

Human Endeavour, a charitable organization from Ontario, Canada, leveraged technology, automation, and intelligence, along with training and support, and offered simplified, individually customized technology.

With a focus on technology to support individuals in completing activities of daily living, tablets were provided to 110 seniors in Ontario and Alberta. The tablets automatically send voice reminders and verification prompts for essential tasks in one of 15 programmed languages. They interact with seniors via voice commands and notify carers through auto‐generated texts and emails when key tasks are missed. A multilingual manual, a call center for help, remote access for debugging, and field upgrades offer real‐time assistance. The tablets also improve safety and social connection through simplified picture‐based calling, mind‐stimulating games, and apps for social engagement. Over 24 months, 11 organizations collaborated to distribute the technology and assess its impact.

**Result:**

Through a community academic partnership, a phase‐1 impact evaluation by York University has been completed. A total of 69 surveys and 21 interviews have been conducted to date. A majority of seniors strongly agreed or agreed that the tablet had a positive impact: 81% said it helped them remember to complete activities of daily living, 87% said it improved health behaviours (e.g. on time medication), 83%/74%/82% said, it helped improved cognitive/emotional/social well‐being, and 79% felt it met their needs. 77% of organizations received positive feedback from clients and 60% of the carers indicated that the quality of life of the senior has improved by having the tablet.

**Conclusion:**

Results from the impact evaluation survey and in‐depth interviews make a compelling case for designing an individually customized technology‐assistive environment for seniors with dementia or MCI. Such a solution supports independence and enables seniors to live longer in the comfort of their own homes.